# Efficacy of a multimodal physiotherapy treatment program for hip osteoarthritis: a randomised placebo-controlled trial protocol

**DOI:** 10.1186/1471-2474-11-238

**Published:** 2010-10-14

**Authors:** Kim L Bennell, Thorlene Egerton, Yong-Hao Pua, J Haxby Abbott, Kevin Sims, Ben Metcalf, Fiona McManus, Tim V Wrigley, Andrew Forbes, Anthony Harris, Rachelle Buchbinder

**Affiliations:** 1 Centre for Health, Exercise & Sports Medicine, School of Health Sciences, University of Melbourne, Melbourne, VIC, Australia; 2 Department of Physiotherapy, Singapore General Hospital, Singapore; 3 Dunedin School of Medicine, University of Otago, Dunedin, New Zealand; 4 Cricket Australia Centre of Excellence, Brisbane, Australia; 5 Department of Epidemiology and Preventive Medicine, School of Public Health and Preventive Medicine, Monash University, Melbourne, VIC, Australia; 6 Centre for Health Economics, Monash University, Melbourne, VIC, Australia; 7 Monash Department of Clinical Epidemiology, Cabrini Hospital, Melbourne, VIC, Australia

## Abstract

**Background:**

Hip osteoarthritis (OA) is a common condition leading to pain, disability and reduced quality of life. There is currently limited evidence to support the use of conservative, non-pharmacological treatments for hip OA. Exercise and manual therapy have both shown promise and are typically used together by physiotherapists to manage painful hip OA. The aim of this randomised controlled trial is to compare the efficacy of a physiotherapy treatment program with placebo treatment in reducing pain and improving physical function.

**Methods:**

The trial will be conducted at the University of Melbourne Centre for Health, Exercise and Sports Medicine. 128 participants with hip pain greater or equal to 40/100 on visual analogue scale (VAS) and evidence of OA on x-ray will be recruited. Treatment will be provided by eight community physiotherapists in the Melbourne metropolitan region. The active physiotherapy treatment will comprise a semi-structured program of manual therapy and exercise plus education and advice. The placebo treatment will consist of sham ultrasound and the application of non-therapeutic gel. The participants and the study assessor will be blinded to the treatment allocation. Primary outcomes will be pain measured by VAS and physical function recorded on the Western Ontario and McMaster Universities Osteoarthritis Index (WOMAC) immediately after the 12 week intervention. Participants will also be followed up at 36 weeks post baseline.

**Conclusions:**

The trial design has important strengths of reproducibility and reflecting contemporary physiotherapy practice. The findings from this randomised trial will provide evidence for the efficacy of a physiotherapy program for painful hip OA.

**Trial Registration:**

Australian New Zealand Clinical Trials Registry reference: ACTRN12610000439044

## Background

Hip osteoarthritis (OA) is a prevalent chronic musculoskeletal condition causing pain, disability, and reduced quality-of-life in affected individuals [1]. There is currently no cure and total hip joint replacement is common for advanced disease. Thus hip OA is a major public health problem contributing to substantial patient morbidity, health care costs and lengthy surgical waiting lists.

While conservative non-pharmacological treatments, such as exercise, are recommended for hip OA [2,3], unlike knee OA, there is little evidence to support their effectiveness. Of the hip OA research, 79% of the published literature involves surgical treatment [4]. This lack of evidence has been highlighted by the European League against Rheumatism (EULAR), a major international rheumatology body [4]. Not surprisingly then, EULAR placed randomised controlled trials of conservative non-drug treatments as one of its top research priorities for hip OA.

A recent systematic review of land-based exercise for hip OA combining the results of five clinical trials demonstrated a small favourable treatment effect for pain, but no benefit in terms of self-reported physical function [5]. Similar conclusions were reached by the authors of another recent systematic review which stated that there was insufficient evidence to suggest that exercise therapy alone can be an effective short-term management approach for reducing pain levels, function, and quality of life [6]. In contrast, the results of a meta-analysis of the benefits of exercise, including water-based programs, for pain relief in hip OA were more favourable [7]. The review concluded that therapeutic exercise, especially with specialised hand-on exercise training and an element of strengthening, is an efficacious treatment for hip OA.

There is evidence from a high quality randomised controlled trial that manual therapy may be more effective than exercise in hip OA [8]. A 5-week manual therapy program comprising mobilisation and manipulation of the hip joint was compared to a therapist-supervised exercise program in 109 patients with hip OA [8]. Both groups showed improvement but the success rate in the manual therapy group (81%) was significantly better than that in the exercise group (50%). Benefits in favour of manual therapy were maintained at a 29-week follow-up.

Physiotherapy treatment can comprise a number of components including exercise, manual therapy, education and advice, and the prescription of gait aids. In practice most or all of these components, tailored to the individual patient's particular presenting musculoskeletal impairments, are provided within physiotherapy treatment programs. While several studies and reviews have evaluated individual components of conservative treatments, none has evaluated a multimodal approach typical of physiotherapy treatment for hip OA. Given the limited research in the area, this project primarily aims to investigate the efficacy of a 12-week multimodal physiotherapy program to treat pain and physical dysfunction in individuals with hip OA. Secondary aims are to assess changes in relevant musculoskeletal impairments with treatment, maintenance of treatment effects over 6 months and the cost-effectiveness of physiotherapy.

### Primary hypothesis

H1: A 12-week multimodal, individualised physiotherapy program will result in significantly greater improvements in pain and physical function than sham physiotherapy immediately post-treatment in individuals with hip OA.

### Secondary hypotheses

H2: A physiotherapy program will result in significantly better participant-perceived response to treatment and greater improvements in health-related quality of life, functional performance, gait biomechanics and musculoskeletal impairments than sham physiotherapy immediately post treatment.

H3: Symptomatic improvements following a 12-week physiotherapy program will be sustained at a 6-month follow-up with a home exercise program.

H4: A physiotherapy program will be more cost-effective than sham physiotherapy when hip OA-related costs are compared and related to the effects of the active intervention.

This paper provides the rationale and background to the study and outlines the design and analysis plan.

## Methods

### Trial design

This is a randomised, assessor- and participant-blinded, placebo controlled trial of a 12-week physiotherapy program with a 6-month follow-up. Measurements will be taken at baseline, 13 weeks and 36 weeks. The protocol conforms to CONSORT guidelines for non-pharmacological interventions [9] and the protocol is designed to conform to the principles of the Declaration of Helsinki.

### Participants

We will recruit 128 people from the community in the Melbourne metropolitan region via advertisements in local clubs, libraries, print and radio media, and Facebook, and from medical practitioners (orthopaedic surgeons, rheumatologists and general practitioners). We will also use our database of people who were recruited from the community for prior studies and have given consent for future contact.

To be eligible, participants must have:

(i) hip OA fulfilling American College of Rheumatology classification criteria [10] with pain in the groin or hip region on most days of the past month and femoral or acetabular osteophytes and joint space narrowing (superior, axial and/or medial) ≥ Grade 2 on a standing x-ray;

(ii) overall average hip/groin pain in the last week ≥ 40 on 100 mm visual analogue scale (VAS) (to ensure a minimal level of pain);

(iii) pain in the groin or hip region for more than 3 months;

(iv) moderate level of interference in activities of daily living;

(v) aged ≥ 50 years.

The exclusion criteria are:

(i) hip surgery within past 6 months;

(ii) awaiting or planning any back or lower limb surgery in the next 9 months;

(iii) current or past (within 3 months) oral or intra-articular corticosteroid use;

(iv) systemic arthritic conditions such as rheumatoid arthritis;

(v) history of hip or knee joint replacement or osteotomy on the test leg;

(vi) other previous hip pathology such as fracture or cancer on the test leg;

(vii) other muscular, joint or neurological condition causing pain or affecting lower limb function;

(viii) physiotherapy, chiropractic treatment or exercises specifically for the hip or lumbar spine in past 6 months;

(ix) any medical or physical impairment apart from hip OA precluding safe participation in exercise or manual therapy such as uncontrolled hypertension, or morbid obesity (body mass index > 40);

(x) walking continuously for more than 30 minutes daily or participating in exercise more than once a week;

(xi) inability to walk unaided;

(xii) unable to comply with protocol;

(xiii) inadequate written and spoken English.

### Procedure

**Figure 1:**
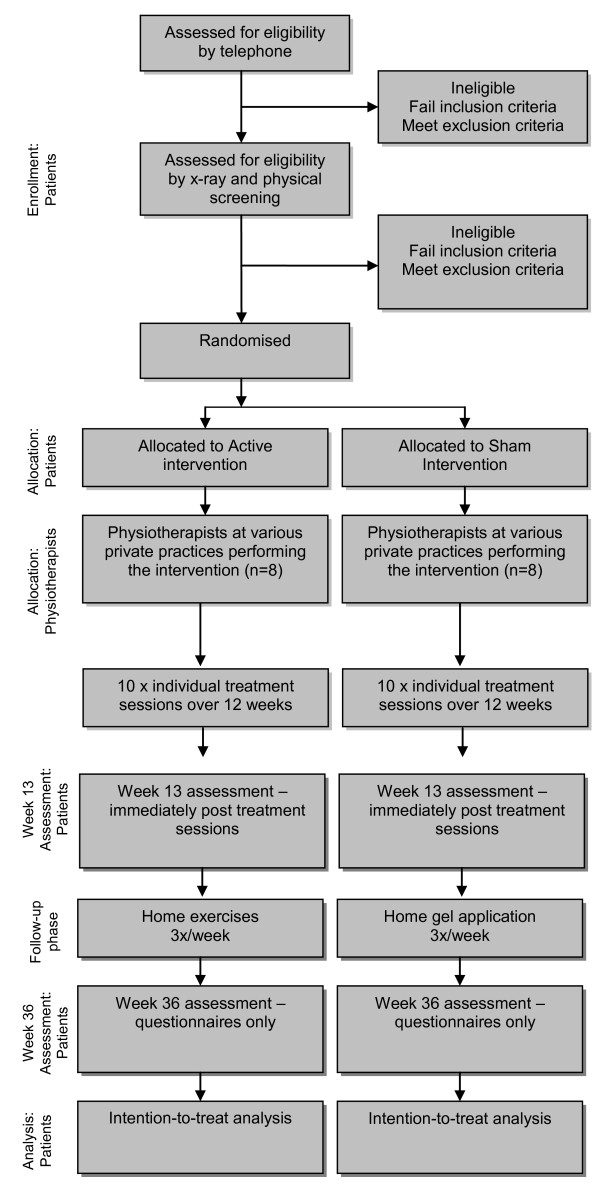
**Flow diagram of study protocol**.

The procedure is outlined in Figure 1. Preliminary screening will be conducted over the telephone. A standardised anteroposterior pelvic x-ray will be obtained in a standing position with 15° internal foot rotation [11] at one of three trial radiology centres, unless they can provide their own films from a weight-bearing x-ray within the previous 12 months. X-rays will be used to determine eligibility and to grade OA severity. X-ray grading will be performed by two trained researchers and any disagreement will be resolved through discussion or where necessary, a third rater. Potential participants will then attend the Department of Physiotherapy, University of Melbourne for physical screening by a physiotherapist to ensure that the reported symptoms are arising from the hip and not from the lumbar spine. We will maintain a screening record to document the criteria eliminating those found to be ineligible. Following baseline testing, the participant will be randomised into one of two groups: (i) active physiotherapy or (ii) sham physiotherapy. Participants will be reassessed after the 12-week intervention (week 13) and again six months later (week 36). During the 6 month follow-up period, participants in the active physiotherapy group will be requested to continue with an unsupervised home exercise program while those in the sham physiotherapy group will be asked to gently apply non-therapeutic gel to their hip region at home. Participants will be asked to refrain from seeking other treatments during the trial but analgesia and anti-inflammatory drugs will be permitted. All medication use and co-interventions will be recorded.

Ethical approval has been obtained from the University of Melbourne Human Research Ethics Committee (HREC #0932812) and Radiation Safety Human Services. All participants will provide written informed consent.

### Randomisation and allocation concealment

The randomisation schedule will be prepared by the study biostatistician (AF) using a computer generated random numbers table. Randomisation will be by random permuted blocks stratified according to therapist (to control for therapist variation). To conceal randomisation, consecutively numbered, sealed, opaque envelopes will be used and maintained centrally. An independent staff member will prepare the envelopes. These will be kept in a locked location accessible only by a research administrator. An envelope will be opened in sequence once the participant has completed all baseline measurements. Allocation will be revealed to the treating physiotherapist by fax/email prior to the participant presenting for treatment.

### Blinding

The outcome assessor (BM) will be blind to group allocation and will not be involved in providing the interventions nor will s/he visit any of the treatment centres. The plain language statement will inform participants that they have an equal chance of receiving active or sham physiotherapy but it will not provide any details of the actual treatments. To assist with blinding, participants in different groups will not attend treatment sessions concurrently. Participants will be requested not to disclose details about their treatment with the outcome assessor. The physiotherapists delivering the interventions cannot be blinded. The statistician(s) performing the statistical analyses will be blind to group allocation until completion of the analyses.

### Interventions

Participants in both groups will attend 10 individual treatment sessions with a physiotherapist over 12 weeks (Table 1). The timing of the sessions will be twice in the first week then weekly until week 7, then two weeks apart for the final two appointments. The first two treatment sessions will last for 45-60 minutes to allow for a more detailed subjective and objective assessment, after which all sessions will be 30 minutes in duration. This reflects a realistic treatment dosage in clinical practice.

**Table 1:** Overview of Active Physiotherapy and Sham Physiotherapy Treatments. The times given are approximate

Active Physiotherapy Overview	Sham Physiotherapy Overview
**Session 1 (45-60 mins)** • Subjective and objective assessment (20-25 mins) • Patient education (5 mins) • 2 of the mandatory manual therapy techniques (5-10 mins) • Teach hip abduction and knee extension strengthening home exercises (15-20 mins)	**Session 1 (45-60 mins)** • Subjective and objective assessment (20-25 mins) • Patient education (5 mins) • Information about gel and application (5-10 mins) • Information about pulsed ultrasound and application (10 mins) • Discuss log-book and attendance (5-10 mins)

	

**Session 2 (45-60 mins)** • Subjective and objective re-assessment (10 mins) • Patient education (5-10 mins) • 2-4 of the mandatory manual therapy techniques (15-20 mins) • Teach all mandatory home exercises and check log-book (15-20 mins)	**Session 2 (45-60 mins)** • Subjective and objective re-assessment (23 mins) • Application of sham gel (10-15 mins) • Application of sham pulsed ultrasound (10-15 mins) • Check and discuss log-book (2-7 mins)

	

**Session 3-10 (30 mins)** • Subjective and objective re-assessment (5 mins) • All mandatory manual therapy techniques with addition of optional techniques if justified (15 mins) • Progress all mandatory home exercises, addition of optional exercises if justified and check log-book (10 mins)	**Session 3-10 (30 mins)** • Subjective and objective re-assessment (13 mins) • Application of sham gel (7 mins) • Application of sham pulsed ultrasound (8 mins) •Check log-book (2 mins)

	

**Follow-up period** • 5 home exercises 3 times/week	**Follow-up period** • Self-application of gel 3 times/week

Eight experienced Project Physiotherapists (at least 5 years of relevant clinical experience and with postgraduate qualifications in manipulative or musculoskeletal physiotherapy) located at private practices in metropolitan Melbourne will be trained to deliver both the active and sham treatments. The training involves a 2-day training course delivered by experienced clinician researchers (JHA, KS and KB) together with a detailed 100-page treatment manual and a DVD showing the techniques and exercises. Having a number of treating physiotherapists is necessary for practical reasons and to improve the generalisability of results. To ensure that factors such as therapist personality and style, which may influence outcome, are evenly distributed between treatment groups, each therapist will treat similar proportions of active and sham participants. At the end of each treatment session, the therapist will complete a 'Treatment Notes' form to record the interventions administered.

The integrity of the interventions will be ensured by a variety of methods. Therapist adherence to the protocol will be ensured by holding training sessions and regular therapist team meetings, providing a comprehensive treatment manual and DVD, having structured recording forms, auditing the recording forms, observing treatment sessions and interviewing participants. To evaluate the credibility of both interventions, the Treatment Credibility Scale [12] will be administered by the therapists to participants after the first and last physiotherapy treatment sessions.

#### Active physiotherapy

The active physiotherapy program is semi-structured comprising core components plus optional additional treatment components. The therapist is able to choose the most appropriate combination and level of manual therapy techniques and exercises from a limited, pre-specified range. Initial selections are based on a standardised clinical assessment performed in the first session. The clinical assessment includes subjective and objective components following which the physiotherapist will generate a problem list and identify the musculoskeletal impairments to be the treatment priorities. The objective assessment includes functional movements, passive range of motion and muscle length tests, muscle strength tests and lumbar-pelvic control tests. A brief subjective and objective assessment will also be performed at the start of each subsequent treatment session to gauge response to treatment, re-identify treatment priorities and alter selection of techniques and exercises.

The four treatment components are (Table 2):

**Table 2:** Details of the Physiotherapy Treatment

Manual Therapy Techniques	Description	Dosage
*Mandatory techniques:*		

Long axis distraction with thrust	Supine. The hip is in 15-30° flexion, 15-30 ° AB, slight ER. Preferably use seat belt. Perform 3-6 thrusts at the beginning of the first set then perform oscillations in the remaining sets.	3-6 sets of 30 secs
Seatbelt glide, or distraction mobilisations, with the hip flexed	Supine with hip flexed and using a seatbelt, oscillatory passive accessory mobilisation forces applied caudally or laterally to the proximal thigh.	3-6 sets of 30 secs
Internal rotation in prone	Prone with knee flexed. IR until contralateral pelvis rises, apply oscillatory force downwards to contralateral pelvis.	3-6 sets of 30 secs
Soft-tissue or deep-tissue massage of quads, adductors, hamstrings, psoas, lateral hip muscles and/or posterior hip muscles and associated fascia	Firm effleurage stroke, deep frictions or sustained pressure trigger point release with the muscle on stretch.	2-5 mins

*Optional techniques:*		

Long axis distraction in prone	Prone. The hip is in 10-15 ° AB. Preferably use seat belt. Perform caudally directed oscillations. May perform 3-6 thrusts at the beginning of the first set.	3-6 sets of 30 secs
Antero-posterior progression (posterior glide)	Supine with hip in flexion and adduction. Use body weight to impart passive oscillations to the postero-lateral hip capsule through the long axis of the femur. Add more flexion, adduction, &/or internal rotation to progress.	3-6 sets of 30 secs
Postero-anterior progression (anterior glide)	Prone with knee bent. Leg supported at knee (may use seatbelt). Pressure applied inferior and medial to greater trochanter in posterior to anterior direction. Vary amount of hip flexion/extension, AB/AD, IR/ER. Modify to use figure-4 position and apply pressure through sacrum.	3-6 sets of 30 secs
Manual stretches to one joint knee extensors, rectus femoris, hip flexors, hamstrings, hip internal rotators, hip external rotators, or hip adductors	Stretch should be felt in target muscle. Manual stretches should match the soft or deep tissue massage technique selected.	6 reps × 20 secs or 4 × 30 secs or 2 × 60 secs
Lumbar spine mobilisation	- Unilateral postero-anterior accessory glides - Passive physiological lumbar spine rotation - Lumbar spine manipulation (supine) - Lumbar spine manipulation (side lying with affected hip up)	3-6 sets of 30 secs

**Home exercises**	**Description**	**Dosage**

*Mandatory techniques:*	*[Exercise difficulty must be progressed]*	

Hip abductor strengthening	Progressed through supine, standing, side lying and standing wall press.	3 × 10 repetitions
Quads strengthening exercise	Progressed through sitting elastic band press or knee extension, partial squats, partial wall squats*, sit-to stand* and split sit to stand.	3 × 10 repetitions
Muscle stretch (should match with the soft tissue/deep tissue massage technique chosen in the manual therapy section	- Hip extension - Hip flextion - Hip abduction and external rotation - Hip internal rotation	2 mins total with 20-60 sec hold times
Challenging functional neuromuscular balance/gait drills set	- standing weight shifting forwards^#^, lateral^# ^and tandem stance^#^ - standing hip control progressing to eccentric hip abductor exercise - balance in double leg stance on foam^# ^or single leg stance^#^ - side stepping progressing to 'carioca' (or braiding) - shuttle walking - stairs	1-2 exercises (4 minutes total time)

		

*Optional techniques:*	*[Maximum of two]*	

Strengthening exercise(s)	- hip extensors progressed through gluteal sets, bridging* and unilateral bridging - hip external rotators progressed through clamshells (or sitting or 4-point kneeling internal rotation), resisted clamshells (or sitting internal rotation) and standing wall press - hip internal rotators progressed through 4-point kneeling, sitting and bottom-leg clamshells	3 × 10 repetitions
Additional stretches(s)	(as above)	
Lumbopelvic control exercise	Supine pelvic tilt with progressions	10 repetitions

	* Option to add elastic band resistance around both knees. ^# ^Option to close eyes.	

**Patient education**		

- About osteoarthritis - Response to exercise and daily physical activity - Activity-rest cycle versus over-activity cycle - Joint protection advice		

**Prescription of a single walking stick**	Only if it will enable the patient to improve their gait pattern and increase their daily physical activity.	

**Stationary cycling**	Up to 10 minutes at a moderate level of intensity (rated as "hard" up to "very hard" - level 5-8 on the Modified Borg Rating Scale of Perceived Exertion achieved within 2 minutes of activity if possible) after each treatment session while at the physiotherapy clinic.	

The treatment program was structured to include a number of mandatory components plus some optional components. Individual technique selection was guided by assessment findings and radiological presentation.

AB = abduction, AD = adduction, ER = external rotation, IR = internal rotation

(i) Manual therapy techniques applied by the therapist and designed to improve the quality and range of motion of the hip and surrounding soft tissues and to reduce pain.

(ii) Home exercises and 10 minutes cycling on a stationary exercise bike at the end of each physiotherapy treatment session.

(iii) Education and advice covering issues such as what is OA, why does it occur, how it is treated, what are the benefits of exercise, the importance of increasing overall physical activity levels in everyday life and how to protect the joint during activities such as sitting, walking, stair climbing, standing, load-carrying and sleeping.

(iv) Provision of a gait aid (single walking stick) to be used in the contralateral hand if deemed necessary by the therapist to improve gait pattern and reduce pain response to walking.

The participant will perform the prescribed home exercises four times weekly, for which weights and resistance elastic bands will be provided as well as hand-outs describing the exercises. To assist with adherence, the number of home exercises is limited to between four and six [13]. A log-book will be completed by participants to record adherence to the home exercises during the treatment period.

#### Sham physiotherapy

As for many procedural interventions, it is difficult to design a placebo treatment that completely mimics a physiotherapy program. However, our aim is to control for attention and time with a therapist and the belief that treatment will assist hip OA. Participants in the sham physiotherapy group will receive the same number and length of visits as those in the active physiotherapy group but will receive only inactive ultrasound and gentle application of a non-therapeutic gel. These emulate common physiotherapy modalities and thus represent realistic placebo options. Participants in the sham group will receive no instruction in exercise techniques and no manual therapy. We have used this identical sham protocol in four randomised controlled trials of physiotherapy for patellofemoral pain [14], knee OA [15], chronic shoulder pain [16] and frozen shoulder [17] involving more than 580 participants. On average 50%-70% of those in the sham group thought they received 'real' treatment or were unsure, and formal statistical analysis showed that blinding was successful. Furthermore, a similar sham protocol was used as a control for exercise in a low back pain trial and participants rated the credibility of this sham treatment highly [18]. This indicates that this sham treatment provides acceptable blinding in a trial of this nature.

### Follow-up period

During the 6 month follow-up period, participants in the active physiotherapy group will perform an unsupervised home exercise program prescribed by their physiotherapist at their final treatment session. The program will comprise five exercises performed three times per week. Participants in the sham physiotherapy group will gently apply the non-therapeutic gel to their hip region for five minutes, three times per week.

### Outcome measures

Outcome measures have been selected based on those recommended for clinical trials of OA [19,20]. In those with bilateral hip OA, only the most symptomatic hip will be assessed.

Age, gender, duration of hip OA symptoms, previous treatment, surgery and medication use for hip OA, employment status, marital status, education level and previous health problems will be obtained by questionnaire. Radiographic disease severity will be assessed from the baseline x-ray using the Kellgren and Lawrence grading system [21] while individual features of osteophytes and joint space narrowing will be rated using the Osteoarthritis Research Society International Grading system [22].

#### Primary outcome measures

The primary outcomes will be change in pain and change in self-reported physical function at 13 weeks (Table 3). Overall average hip pain in the past week will be self-assessed by a 100 mm VAS with terminal descriptors of 'no pain' and 'worst pain possible'. Such measurement has demonstrated reliability in OA [19]. Physical function will be self-assessed using the Western Ontario and McMaster Universities Osteoarthritis Index (WOMAC) Likert version 3.1. This is a disease-specific instrument whose validity, reliability and responsiveness have been demonstrated in an extensive range of OA studies [23]. The physical function subscale has 17 items with a five point Likert response giving a total score out of 68.

**Table 3:** Summary of measures to be collected

Primary outcome measures	Data collection instrument	Collection points
Average pain in past week	100 mm VAS	0, 13, 36 weeks
Physical function in past 48 hours	WOMAC Osteoarthritis Index 3.1 Likert version	0, 13, 36 weeks

**Secondary outcome measures**		

Pain, function, and stiffness	HOOS (incorporating WOMAC)	0, 13, 36 weeks
Health-related quality of life	Assessment of Quality of Life Instrument version 2 (AQoL II)	0, 13, 36 weeks
Patient's global rating of change overall and for pain and function	7-point ordinal scale	13, 36 weeks
Self efficacy	Arthritis Self-efficacy scale	0, 13, 36 weeks
Pain catastrophizing	Pain Catastrophizing Scale	0, 13, 36 weeks
Coping strategies	Coping Strategies questionnaire	0, 13, 36 weeks
Objective functional performance	Timed 40 m walk Timed stair climb (ascent and descent) 30 second sit-to-stand	0, 13 weeks
Standing balance	Step test 4-square step test	0, 13 weeks
Hip range of motion	Clinical methods and inclinometer	0, 13 weeks
Hip and knee muscle strength	Isometric - isokinetic dynamometer (quadriceps and hamstrings) and instrumented manual muscle tester (hip flexors, extensors, abductors, rotators)	0, 13 weeks
Gait biomechanics (in a subset)	3-dimensional motion analysis system	0, 13 weeks

**Other measures**		

Physical activity levels	Physical Activity Scale for the Elderly (PASE) Pedometer worn for 7 days	0, 13, 36 weeks
Treatment credibility	Treatment Credibility Scale	1,12 weeks
Participant success of blinding	Questionnaire	13, 36 weeks
Healthcare consumption and related costs	Questionnaire; health system records	0, 5, 9, 13, 36 weeks
Adverse events	Participant log-book	Throughout
Adherence - treatment session attendance; home exercise or gel application	Participant log-book; Therapist treatment records; Questionnaire	Throughout

#### Secondary outcome measures

A number of secondary measures will be included (Table 3). The Hip Osteoarthritis Outcome Scale (HOOS) is a patient-administered measure that assesses the patient's opinion of their hip and associated problems over the previous week. It consists of five subscales; pain, other symptoms, function in daily living, function in sport and recreation, and hip-related quality of life. A normalised score (100 indicating no symptoms and 0 indicating extreme symptoms) is calculated for each subscale [24].

A number of functional performance tests and musculoskeletal impairments will be measured at baseline and at 13 weeks. The passive range of hip flexion, extension, abduction, internal rotation and external rotation will be measured using clinical methods and an inclinometer. The test retest reliability ICCs for these measures ranged from 0.82 to 0.94 in 25 patients with hip OA tested one week apart [25]. For all measures, two trials will be performed and the mean reading used in analysis. Hip flexion, extension and abduction will be measured in supine while internal and external rotation will be measured in sitting

An instrumented manual muscle tester will be used to measure maximum, normalised isometric strength (peak torque; Nm/kg) of the hip abductor, extensor, flexor and internal and external rotator muscles. Our reliability ICCs ranged from 0.65 to 0.85 in six patients with hip OA tested a week apart [25]. Measurements of hip abductors and extensors will be taken in supine while those of the flexor and rotator muscles will be taken in sitting. Maximum voluntary isometric torque of the quadriceps and hamstring muscles at 60° knee flexion will be measured in sitting using a KinCom isokinetic dynamometer (reliability ICCs were 0.85 for quadriceps and 0.85 for hamstrings for ten patients with painful hip OA). Testing will comprise two maximal contractions with the peak value used for analysis.

Several functional tests will be included. The stair climb test [26] involves timing how long it takes participants to ascend and descend six steps at their own pace. For the 30 second sit-to-stand test, the number of times participants can rise to a full standing position from sitting and return to sitting in 30 seconds is counted [27]. Walking performance will be assessed by calculating walking velocity (m/sec) as participants walk 20 meters with the instructions 'walk as quickly as you can without overexerting yourself' [28]. Dynamic standing balance will be assessed by the step test [29] and the 4-square step test [30].

In a subset of participants, gait analysis will be performed at baseline and at 13 weeks. Kinematic and ground reaction force data will be recorded simultaneously for five walking trials in usual footwear at self-selected pace using a 3-D motion analysis system with12 cameras (Vicon MX, Oxford, UK) and three force plates (AMTI, Massachusetts, USA) concealed in the floor. Kinematics will be derived from the standard Davis-Kadaba marker set using Vicon Plug-in Gait model. Data will be combined using inverse dynamics to yield measures of external joint moments. The main measures will be peak external hip adduction and abduction moments, peak external hip flexion and extension moments, pelvic drop/rise in the frontal plane and pelvic rotation ranges of motion, and peak and total range of hip motion in the sagittal and transverse planes. Reliability in our laboratory is good for the measures we have subjected to reliability testing, with ICCs of 0.56 to 0.95 (the majority > 0.80) from six patients with hip OA and six controls tested twice one week apart.

The Assessment of Quality of Life instrument version 2 (AQoL II) has 20 questions that cover six dimensions of health-related quality of life including independent living, social relationships, physical senses, coping, pain and psychological wellbeing. The AQoL has strong psychometric properties and is more responsive than other widely-used scales [31,32]. It produces a single utility index that ranges from -0.04 (worst possible health-related quality of life) to 1.00 (full health-related quality of life). A clinically important difference in health-related quality of life can be defined as a change of 0.04 AQoL units [33]. The AQoL will be collected at weeks 0, 13, and 36.

Habitual physical activity will be measured in two ways, using a questionnaire and using a pedometer. The Physical Activity Scale for the Elderly (PASE) will be used to measure both the level and type of recreational and occupational physical activity undertaken by participants over the previous week. The PASE was developed and validated in samples of older adults (age 55+ years) [34]. A pedometer (HJ-005 Omron Healthcare, Japan) will be worn for a week on three occasions (baseline, 13 weeks and 36 weeks) to record the number of steps taken per day. Participants will be asked to wear the pedometer full time during their waking hours. Pedometers have been found to be a simple and inexpensive means to estimate physical activity levels [35,36]. It is recommended that at least three days of sampling are needed to accurately assess activity levels given differences between weekends and weekdays [37].

The Arthritis Self Efficacy Scale will be used to measure psychological status. It has three subscales that assess self-efficacy for control of pain management, physical function and other arthritis symptoms. Prior studies have supported both the reliability and validity of this scale [38]. Pain catastrophizing will be measured using the 13-item Pain Catastrophizing Scale. It measures tendencies to ruminate about pain, magnify pain, and feel helpless about pain. It has high internal consistency (coefficient alpha = .87) and is associated with heightened pain, psychological distress, and physical disability [39]. We will use the Coping Attempts Scale of the Coping Strategies Questionnaire to assess the use of pain coping skills [40]. This 42-item scale measures how often a patient engages in 7 different pain coping strategies. This instrument has demonstrated sensitivity to change from treatment in chronic pain samples as well as good internal consistency and construct validity [41].

Participants will rate their perceived overall change and their change specifically in pain and in physical function with treatment (compared to baseline) on seven-point ordinal scales (1-much worse to 7-much better). Scales of this kind are frequently used as an external criterion for comparison with changes in scores of other outcomes [42]. Measuring patient perceived change using a rating of change scale has been shown to be a clinically relevant and stable concept for interpreting truly meaningful improvements from the individual perspective [43]. We will also dichotomise the group according to their perceived change rating where *improved *will be defined as 'moderately' or 'much' better, and *not improved *will be defined as 'slightly' better and below. The proportion of improved participants from each group will determine success of the treatment.

#### Other measures

A number of other measures will be obtained (Table 3). Participant adherence will be obtained by recording the number of physiotherapy sessions attended (out of a maximum number of ten). Those in the active physiotherapy group will complete a daily log-book to record the number of home exercise sessions completed during the treatment phase. To indicate adherence to the home program during the six-month follow-up period, participants in both groups will be mailed a short questionnaire at Weeks 24 and 36 which asks how many times in the past week they have performed the home exercises or applied the gel. Adverse events and the use of co-intervention will be recorded in a log-book and by open-probe questioning by the assessor at trial completion. At the 13 week and 36 week measurement time points, study participants will be asked to indicate which treatment they believe they have received (active or sham) and reasons for that choice to assess the success of blinding.

Information on health care costs and direct non-health care costs over the last month will be collected retrospectively at week 0, 5, 9, 13, and 36 by questionnaire. Direct health care costs will include costs of physiotherapy attendance (assumed zero in the sham group), additional health provider visits (doctors, specialists, other health care professionals), investigative procedures, purchase of prescription and over the counter medication, and hospitalisation. These will be valued using published prices for medical costs. Direct non-health care resources will include number of lost days from work.

The monetary valuation of health status pre- and post-treatment is potentially a more comprehensive patient relevant measure of treatment gains than measures of either a clinical outcome or health-related quality of life. A simple open-ended questionnaire will ask participants in each group about their willingness to pay for the treatment given the outcomes they experience.

### Sample size

Our two primary endpoints are hip pain measured on a VAS and WOMAC physical function score. The minimum clinically important difference to be detected in OA trials is a change in pain of 18 mm (on 100 mm VAS) [44] and a change of six physical function WOMAC units (out of 68) [45]. Based on our previous data, we assume a common between-subject standard deviation of change of 30 mm for pain VAS and 12 units for WOMAC physical function as well as a baseline to 13 weeks correlation in scores of 0.6. These statistics indicate a smaller standardised effect size of interest (Cohen's *d*) of 0.5 for the WOMAC measure than the *d *of 0.6 for pain. The required sample for an analysis of covariance of change in scores controlling for the baseline value of the variable when *d *is 0.5, power is 0.9 and type I error is set at .05 is 54 participants per group. In addition, at 54 participants per group the power will be even greater for pain. Allowing for an approximate 15% drop-out rate, we will recruit 64 participants per group.

### Data and statistical analysis

The primary analysis of the data will be undertaken using the principle of intention-to-treat (ITT). Our ITT analysis will include all participants including those who have missing data and those who are not fully compliant with the protocol. Some attrition is anticipated despite the fact that we will implement procedures to minimise loss to follow-up and participant withdrawal, and maximise adherence. Multiple imputation methodology will be employed to account for missing data [46].

Demographic and clinical characteristics as well as baseline data will be presented to assess the baseline comparability of the intervention groups. These variables will also be examined for those participants who withdraw from the study and those who remain.

Descriptive statistics will be presented for each group as the mean change (standard deviation, 95% confidence intervals) in the outcomes from baseline to each time point. Differences in mean change from baseline to each time point will be compared between groups using generalised linear regression modelling adjusting for baseline levels of the outcome measure. Model assumptions will be checked by standard diagnostic plots.

Improvement between active and sham physiotherapy groups based on the perceived ratings of change will be compared using log binomial regression and presented as relative risks with 95% confidence intervals.

As part of the secondary analyses of the primary outcomes at 13 and 36 weeks, we plan to conduct a separate analysis to estimate the effect of treatment in the hypothetical scenario of full adherence to randomised treatment modality. Adherence will be defined as attendance at more than 80% of scheduled treatment sessions and 60% of prescribed home exercise sessions in the active physiotherapy group. This is based on definitions used in another study of manual therapy and exercise in hip OA [47]. Analytical methods for this will utilise instrumental variables methodology involving two stage least squares estimation [48].

An index (with bootstrap 95% confidence intervals) to assess the success of participant blinding will be computed [49].

No statistical adjustment will be made for multiple testing. All tests will be two sided and carried out at the 5% level of significance. Any changes to the study design or analysis plan will be documented with full justification.

### Economic evaluation

The economic evaluation will be conducted from the perspective of the Australian health care system and the individual patient. The primary economic evaluation will take the form of a cost effectiveness study of the cost per extra quality adjusted life years (QALYs). QALYs will be calculated using the AQoL scores over 36 weeks. Differences in mean change from baseline for the AQoL to each time point will be weighted by the time from baseline using generalised linear regression modelling adjusting for baseline levels of the AQoL to construct QALYs, and then compared between groups. Differences in the mean cost between groups will be calculated using generalised linear regression modelling. Incremental cost per QALY will be calculated as the ratio of the difference in mean cost to the difference in mean QALYs with 95% confidence intervals calculated using Fieller's theorem. As supplementary analyses the incremental cost per extra person with a clinically significant improvement in function, and per extra person perceived to be recovered will be calculated

### Timelines

Ethics approval was obtained from the Human Research Ethics Committee of the University of Melbourne in April 2010. Recruitment and training of the physiotherapists was undertaken in April 2010 and recruitment of participants has commenced. All participants are expected to have completed the study by September 2012.

## Discussion

There are several major strengths of the intervention design in this study. Firstly, the multimodal nature of the active physiotherapy program mimics contemporary physiotherapy clinical practice in Australia and many other countries. Patients are treated with a combination of manual therapy techniques and complementary exercises. Given that both exercise and manual therapy alone have been shown to be effective for symptomatic relief in hip OA [8], we contend that the multimodal nature of our program is likely to be more efficacious than either intervention alone.

Secondly, patients' treatment programs are individualised using a problem-solving approach following assessment of musculoskeletal impairments. This allows the program to target the presenting musculoskeletal impairments that contribute the patients' symptoms and functional limitations. It also aligns with the guidelines from EULAR, which recommend tailored treatment [4]. The semi-structured nature of the program, where there are constraints on the number and options for manual therapy techniques and exercises from which the physiotherapists can choose, will reduce treatment variation and allow the delivered treatments to be more easily reported and replicated. Finally, the delivery of the interventions by multiple practicing community physiotherapists will improve the generalisability of the findings.

We considered it important to control for non-specific treatment effects (often referred to as placebo effects) in the study design, given the role these play in influencing treatment outcome. A recent meta-analysis showed that for active treatment of chronic pain conditions (not specifically hip OA), spontaneous recovery contributes around 10% and placebo effects around 30% [50]. Placebo effects have also been found to be greatest for non-pharmacological interventions and for patient-reported outcomes particularly pain [51]. In fact, the sham physiotherapy treatment that we will utilise was associated with a 38% reduction in pain in our previous study in patients with knee OA [15]. While we acknowledge that such indirect treatment effects are an important component of a physiotherapy treatment, we wish to evaluate the additional benefit of direct treatment that target identified musculoskeletal impairments. We did not include a 'no treatment' option as a third study arm as natural recovery is unlikely to occur in these participants who have at least a mild degree of radiographic joint change and report functional disability at baseline.

Our outcome measures are those recommended for use in clinical trials of OA [20]. These include self-report measures of pain, function, quality of life and global response to treatment. A range of other measures are included to also incorporate functional performance, strength, range of motion, psychological aspects, gait biomechanics and physical activity levels in our investigation. We have specified two primary outcomes with the other measures being either secondary outcomes or mechanistic measures which will help us understand the underlying mechanisms explaining changes in pain and function. A health economics assessment is included given the need to justify the cost-effectiveness of treatments in the current economic climate.

## Conclusions

This study uses a randomised controlled trial design to investigate whether a multimodal individualised physiotherapy program that incorporates manual therapy and exercise is more efficacious and cost-effective for the management of symptoms in people with hip OA than a sham physiotherapy treatment that controls for therapist attention. The study design leads to some limitations of generalisability due to a number of inclusion/exclusion criteria, however the design has major strengths related to reproducibility and reflecting contemporary clinical practice. The findings will enable evidence-based recommendations as to the usefulness of this conservative option for the management of patients with hip OA.

## Competing interests

The authors declare that they have no competing interests.

## Authors' contributions

KLB conceived the project and is leading the co-ordination of the trial. KLB, RB, TW, YHP, AF and AH assisted with protocol design and procured the project funding. KLB, RB and TE wrote this manuscript. AF designed the statistical analysis and provided the randomisation schedule. AH designed the economic evaluation. TW and YHP designed the biomechanical and physical impairment measures. KLB, JHA, KS and YHP designed the active physiotherapy intervention and JHA, KS and KLB trained the therapists. TE wrote the protocol manual and the final drafts of this manuscript. BM is the blinded assessor on the project while FM recruits and screens the participants. All authors participated in the trial design, provided feedback on drafts of this paper and read and approved the final manuscript.

## Pre-publication history

The pre-publication history for this paper can be accessed here:

http://www.biomedcentral.com/1471-2474/11/238/prepub

## References

[B1] Lawrence RC Felson DT Helmick CG Arnold LM Choi H Deyo RA Gabriel S Hirsch R Hochberg MC Hunder GG Estimates of the prevalence of arthritis and other rheumatic conditions in the United States. Part II Arthritis & Rheumatism 2008 58 1 26 35 10.1002/art.23176 PMC3266664 18163497

[B2] Zhang W Moskowitz RW Nuki G Abramson S Altman RD Arden N Bierma-Zeinstra S Brandt KD Croft P Doherty M OARSI recommendations for the management of hip and knee osteoarthritis, Part II: OARSI evidence-based, expert consensus guidelines Osteoarthritis & Cartilage 2008 16 2 137 10.1016/j.joca.2007.12.013 18279766

[B3] Conaghan PG Dickson J Grant RL Care and management of osteoarthritis in adults: summary of NICE guidance BMJ 2008 336 7642 502 503 10.1136/bmj.39490.608009.AD 18310005 PMC2258394

[B4] Zhang W Doherty M Arden N Bannwarth B Bijlsma J Gunther KP Hauselmann HJ Herrero-Beaumont G Jordan K Kaklamanis P EULAR evidence based recommendations for the management of hip osteoarthritis: report of a task force of the EULAR Standing Committee for International Clinical Studies Including Therapeutics (ESCISIT) Ann Rheum Dis 2005 64 5 669 681 10.1136/ard.2004.028886 15471891 PMC1755499

[B5] Fransen M McConnell S Hernandez-Molina G Reichenbach S Exercise for osteoarthritis of the hip Cochrane Database of Systematic Reviews 2009 3 10.1002/14651858.CD007912 19588445

[B6] McNair PJ Simmonds MA Boocock MG Larmer PJ Exercise therapy for the management of osteoarthritis of the hip joint: a systematic review Arthritis Res Ther 2009 11 3 R98 10.1186/ar2743 19555502 PMC2714154

[B7] Hernandez-molina G Reichenbach S Zhang B Lavalley M Felson D Effect of therapeutic exercise for hip osteoarthritis pain: results of a meta-analysis Arthritis Care and Research 2008 59 1221 1228 10.1002/art.24010 18759315 PMC2758534

[B8] Hoeksma H Dekker J Ronday H Heering A van der Lubbe N Vel C Breedveld F van den Ende C Comparison of manual therapy and exercise therapy in osteoarthritis of the hip: a randomized clinical trial Arthritis & Rheumatism (Arthritis Care & Research) 2004 51 5 722 729 10.1002/art.20685 15478147

[B9] Boutron I Moher D Altman DG Schulz KF Ravaud P Extending the CONSORT statement to randomized trials of nonpharmacologic treatment: explanation and elaboration Ann Intern Med 2008 148 4 295 309 18283207 10.7326/0003-4819-148-4-200802190-00008

[B10] Altman DG The American College of Rheumatology criteria for the classification and reporting of osteoarthritis of the hip Arthritis & Rheumatism 1991 34 505 514 10.1002/art.1780340502 2025304

[B11] Altman DG Measurement of structural progression in osteoarthritis of the hip: the Barcelona consensus group Osteoarthritis & Cartilage 2004 12 515 524 10.1016/j.joca.2004.04.004 15219566

[B12] Borkovec T Nau S Credibility of analogue therapy rationales Journal of Behavioural Therapy and Experimental Psychiatry 1972 3 257 260 10.1016/0005-7916(72)90045-6

[B13] Henry KD Rosemond C Eckert LB Effect of number of home exercises on compliance and performance in adults over 65 years of age Phys Ther 1999 79 3 270 277 10078770

[B14] Crossley K Bennell K Green S Cowan S McConnell J Physical therapy for patellofemoral pain: a randomized, double-blinded, placebo-controlled trial Am J Sports Med 2002 30 6 857 865 12435653 10.1177/03635465020300061701

[B15] Bennell KL Hinman RS Metcalf BR Buchbinder R McConnell J McColl G Green S Crossley KM Efficacy of physiotherapy management of knee joint osteoarthritis: a randomised, double blind, placebo controlled trial Annals of the Rheumatic Diseases 2005 64 6 906 912 10.1136/ard.2004.026526 15897310 PMC1755542

[B16] Bennell K Wee E Coburn S Green S Harris A Staples M Forbes A Buchbinder R Efficacy of standardised manual therapy and home exercise programme for chronic rotator cuff disease: randomised placebo controlled trial British Medical Journal 2010 8 340 10.1136/bmj.c2756 PMC2882554 20530557

[B17] Buchbinder R Youd JM Green S Stein A Forbes A Harris A Bennell K Bell S Wright WJ Efficacy and cost-effectiveness of physiotherapy following glenohumeral joint distension for adhesive capsulitis: a randomized trial Arthritis Rheum 2007 57 6 1027 1037 10.1002/art.22892 17665470

[B18] Pengel LH Refshauge KM Maher CG Nicholas MK Herbert RD McNair P Physiotherapist-directed exercise, advice, or both for subacute low back pain: a randomized trial Ann Intern Med 2007 146 11 787 796 17548410 10.7326/0003-4819-146-11-200706050-00007

[B19] Bellamy N Osteoarthritis clinical trials: candidate variables and clinimetric properties Journal of Rheumatology 1997 24 4 768 778 9101516

[B20] Bellamy N Kirwan J Boers M Brooks P Strand V Tugwell P Altman R Brandt K Dougados M Lequesne M Recommendations for a core set of outcome measures for future phase III clinical trials in knee, hip, and hand osteoarthritis. Consensus development at OMERACT III J Rheumatol 1997 24 4 799 802 9101522

[B21] Kellgren JH Lawrence JS Radiological assessment of osteo-arthrosis Annals of the Rheumatic Diseases 1957 16 4 494 502 10.1136/ard.16.4.494 13498604 PMC1006995

[B22] Altman RD Gold GE Atlas of individual radiographic features in osteoarthritis, revised Osteoarthritis & Cartilage 2007 15 Supplement A 10.1016/j.joca.2006.11.009 17320422

[B23] McConnell S Kolopack P Davis AM The Western Ontario and McMaster Universities Osteoarthritis Index (WOMAC): a review of its utility and measurement properties Arthritis Rheum 2001 45 5 453 461 10.1002/1529-0131(200110)45:5<453::AID-ART365>3.0.CO;2-W 11642645

[B24] Klassbo M Larsson E Mannevik E Hip disability and osteoarthritis outcome score. An extension of the Western Ontario and McMaster Universities Osteoarthritis Index Scand J Rheumatol 2003 32 1 46 51 10.1080/03009740310000409 12635946

[B25] Pua Y-H Wrigley TW Cowan SM Bennell KL Intrarater Test-Retest Reliability of Hip Range of Motion and Hip Muscle Strength Measurements in Persons With Hip Osteoarthritis Archives of Physical Medicine and Rehabilitation 2008 89 6 1146 10.1016/j.apmr.2007.10.028 18503813

[B26] Rejeski WJ Ettinger WH Jr Schumaker S James P Burns R Elam JT Assessing performance-related disability in patients with knee osteoarthritis Osteoarthritis Cartilage 1995 3 3 157 167 10.1016/S1063-4584(05)80050-0 8581745

[B27] Csuka M McCarty DJ Simple method for measurement of lower extremity muscle strength Am J Med 1985 78 1 77 81 10.1016/0002-9343(85)90465-6 3966492

[B28] Walsh M Woodhouse LJ Thomas SG Finch E Physical impairments and functional limitations: a comparison of individuals 1 year after total knee arthroplasty with control subjects Phys Ther 1998 78 3 248 258 9520970 10.1093/ptj/78.3.248

[B29] Hill KD Bernhardt J McGann AM Maltese D Berkovits D A new test of dynamic standing balance for stroke patients: reliability, validity and comparison with healthy elderly Physiotherapy Canada 1996 48 4 257 262 10.3138/ptc.48.4.257

[B30] Dite W Temple VA A clinical test of stepping and change of direction to identify multiple falling older adults Arch Phys Med Rehabil 2002 83 11 1566 1571 10.1053/apmr.2002.35469 12422327

[B31] Whitfield K Buchbinder R Segal L Osborne RH Parsimonious and efficient assessment of health-related quality of life in osteoarthritis research: validation of the Assessment of Quality of Life (AQoL) instrument Health Qual Life Outcomes 2006 4 19 10.1186/1477-7525-4-19 16556304 PMC1538577

[B32] Osborne RH Hawthorne G Lew EA Gray LC Quality of life assessment in the community-dwelling elderly: validation of the Assessment of Quality of Life (AQoL) Instrument and comparison with the SF-36 J Clin Epidemiol 2003 56 2 138 147 10.1016/S0895-4356(02)00601-7 12654408

[B33] Hawthorne G Osborne R Population norms and meaningful differences for the Assessment of Quality of Life (AQoL) measure Aust N Z J Public Health 2005 29 2 136 142 10.1111/j.1467-842X.2005.tb00063.x 15915617

[B34] Washburn RA Smith KW Jette AM Janney CA The Physical Activity Scale for the Elderly (PASE): development and evaluation J Clin Epidemiol 1993 46 2 153 162 10.1016/0895-4356(93)90053-4 8437031

[B35] Tudor-Locke C Williams JE Reis JP Pluto D Utility of pedometers for assessing physical activity: construct validity Sports Med 2004 34 5 281 291 10.2165/00007256-200434050-00001 15107007

[B36] Tudor-Locke C Williams JE Reis JP Pluto D Utility of pedometers for assessing physical activity: convergent validity Sports Med 2002 32 12 795 808 10.2165/00007256-200232120-00004 12238942

[B37] Tudor-Locke C Burkett L Reis JP Ainsworth BE Macera CA Wilson DK How many days of pedometer monitoring predict weekly physical activity in adults? Prev Med 2005 40 3 293 298 10.1016/j.ypmed.2004.06.003 15533542

[B38] Lorig K Chastain RL Ung E Shoor S Holman HR Development and evaluation of a scale to measure perceived self-efficacy in people with arthritis Arthritis Rheum 1989 32 1 37 44 10.1002/anr.1780320107 2912463

[B39] Osman A Barrios FX Gutierrez PM Kopper BA Merrifield T Grittmann L The Pain Catastrophizing Scale: further psychometric evaluation with adult samples J Behav Med 2000 23 4 351 365 10.1023/A:1005548801037 10984864

[B40] Rosenstiel AK Keefe FJ The use of coping strategies in chronic low back pain patients: relationship to patient characteristics and current adjustment Pain 1983 17 1 33 44 10.1016/0304-3959(83)90125-2 6226916

[B41] Keefe FJ Caldwell DS Queen KT Gil KM Martinez S Crisson JE Ogden W Nunley J Pain coping strategies in osteoarthritis patients J Consult Clin Psychol 1987 55 2 208 212 10.1037/0022-006X.55.2.208 3571674

[B42] Jaeschke R Singer J Guyatt GH Measurement of health status. Ascertaining the minimal clinically important difference Control Clin Trials 1989 10 4 407 415 10.1016/0197-2456(89)90005-6 2691207

[B43] ten Klooster PM Drossaers-Bakker KW Taal E van de Laar MA Patient-perceived satisfactory improvement (PPSI): interpreting meaningful change in pain from the patient's perspective Pain 2006 121 1-2 151 157 10.1016/j.pain.2005.12.021 16472915

[B44] Bellamy N Carette S Ford PM Kean WF le Riche NG Lussier A Wells GA Campbell J Osteoarthritis antirheumatic drug trials. III. Setting the delta for clinical trials--results of a consensus development (Delphi) exercise Journal of Rheumatology 1992 19 3 451 457 1578462

[B45] Angst F Aeschlimann A Stucki G Smallest detectable and minimal clinically important differences of rehabilitation intervention with their implications for required sample sizes using WOMAC and SF-36 quality of life measurement instruments in patients with osteoarthritis of the lower extremities Arthritis Rheum 2001 45 4 384 391 10.1002/1529-0131(200108)45:4<384::AID-ART352>3.0.CO;2-0 11501727

[B46] Molenberghs G Kenward M Missing Data in Clinical Studies 2007 Chichester, UK: John Wiley and Sons Ltd

[B47] Abbott JH Robertson MC McKenzie JE Baxter GD Theis JC Campbell AJ Exercise therapy, manual therapy, or both, for osteoarthritis of the hip or knee: a factorial randomised controlled trial protocol Trials 2009 10 11 10.1186/1745-6215-10-11 19200399 PMC2644684

[B48] Stuart EA Perry DF Le HN Ialongo NS Estimating intervention effects of prevention programs: accounting for noncompliance Prev Sci 2008 9 4 288 298 10.1007/s11121-008-0104-y 18843535 PMC2921838

[B49] James KE Bloch DA Lee KK Kraemer HC Fuller RK An index for assessing blindness in a multi-centre clinical trial: disulfiram for alcohol cessation--a VA cooperative study Stat Med 1996 15 13 1421 1434 10.1002/(SICI)1097-0258(19960715)15:13<1421::AID-SIM266>3.0.CO;2-H 8841652

[B50] Krogsboll LT Hrobjartsson A Gotzsche PC Spontaneous improvement in randomised clinical trials: meta-analysis of three-armed trials comparing no treatment, placebo and active intervention BMC Med Res Methodol 2009 9 1 10.1186/1471-2288-9-1 19123933 PMC2628943

[B51] Hrobjartsson A Gotzsche PC Is the placebo powerless? An analysis of clinical trials comparing placebo with no treatment N Engl J Med 2001 344 21 1594 1602 10.1056/NEJM200105243442106 11372012

